# Plasma Membrane Proteomics Identifies Biomarkers Associated with MMSET Overexpression in T(4;14) Multiple Myeloma

**DOI:** 10.18632/oncotarget.1049

**Published:** 2013-06-26

**Authors:** Zhigang Xie, Jayantha Gunaratne, Lip Lee Cheong, Shaw Cheng Liu, Tze Loong Koh, Gaofeng Huang, Walter P. Blackstock, Wee Joo Chng

**Affiliations:** ^1^ Cancer Science Institute of Singapore, National University of Singapore, Singapore; ^2^ Quantitative Proteomics Group, Institute of Molecular and Cell Biology, Agency for Science, Technology and Research, Singapore; ^3^ Department of Medicine, Yong Loo Lin School of Medicine, National University of Singapore, Singapore; ^4^ Department of Haematology-Oncology, National University Cancer Institute of Singapore, National University Healthy System, Singapore

**Keywords:** SILAC, quantitative proteomics, oncotargets, multiple myeloma

## Abstract

Multiple myeloma (MM) is characterized by recurrent chromosomal translocations. *MMSET*, identified by its fusion to the IgH locus in t(4;14) MM, is universally overexpressed in t(4;14) MM. In order to identify cell surface biomarkers associated with t(4;14) MM for small molecule or antibody based therapies, we knocked down MMSET expression with shRNA and generated a cell line pair from KMS11, a t(4;14) MM cell line. We used quantitative mass spectrometry to identify plasma membrane proteins associated with MMSET overexpression. Using this approach, 50 cell surface proteins were identified as differentially expressed between KMS11 and KMS11/shMMSET. Western blot and flow cytometry analysis indicated SLAMF7 was over-expressed in t(4;14) MM cell lines and down-regulated by MMSET shRNAs. SLAMF7 expression was also confirmed in primary t(4;14) MM samples by flow cytometry analysis. Quantitative RT-PCR and ChIP analysis indicated MMSET might regulate the transcription level of SLAMF7 and be an important functional element for *SLAMF7* promoter activity. Furthermore, SLAMF7 shRNA could induce G1 arrest or apoptosis and reduce clonogenetic capacity in t(4;14) MM cells. Overall, these results illustrated SLAMF7 might be a novel cell surface protein associated with t(4;14) MM. It is potential to develop t(4;14) MM targeted therapy by SLAMF7 antibody mediated drug delivery.

## INTRODUCTION

Recurrent chromosomal translocations are central to the pathogenesis, diagnosis, and prognosis of hematologic malignancies. In the past decade, it has become apparent that approximately 50% of multiple myeloma (MM) harbor recurrent translocations involving the immunoglobulin heavy chain (IgH) locus on chromosome 14q32 [1-3]. The translocation t(4;14)(p16;q32) is one of the most common translocation in MM, affecting 15% of patients, and is associated with very poor prognosis [4]. The t(4;14) translocation leads to the simultaneous overexpression of two genes, *FGFR3* and *MMSET*. FGFR3 has transforming activity *in vitro* and *in vivo*, but approximately 30% of t(4;14) MM patients do not express FGFR3, whereas overexpression of MMSET isoforms is a universal feature of t(4;14) cases [5-7]. Furthermore, the poor prognosis of t(4;14) persists irrespective of FGFR3 expression [7]. These data suggest that MMSET may be the critical oncogene in this translocation. The *MMSET* gene spans 120 kb, consists of 24 exons and undergoes complex alternative splicing. Two major transcripts were identified: type I encodes a protein of 647 amino acids and type II encodes a protein of 1365 amino acids. Both proteins share a common amino terminus [8]. A third transcript initiated within a middle intron of *MMSET* encodes a protein named RE-IIBP [9].

The 1365 amino acid MMSET protein contains a SET domain that is found in many histone methyltransferases (HMTs) and determines their enzymatic activity [10]. Recently MMSET has been shown to have histone methyltransferase activity and knockdown studies have demonstrated that MMSET upregulation contributes to cellular adhesion, clonogenic growth and tumorigenicity [1, 10, 11]. While differentially expressed genes were identified between cases with and without a t(4;14) by using global gene expression microarrays, the t(4;14) MM proteome is unknown [12, 13]. Currently there is no drug targeting MMSET proteins directly. The implications of identifying proteins with MMSET-dependent expression are clear, as they could potentially constitute novel biomarkers or targets for the treatment of MM. Plasma membrane proteins play a pivotal role in regulating cell-cell interaction, recognition, migration, adhesion, and signal transduction [14]. It is noteworthy that many clinical biomarkers and therapeutic targets are cell surface proteins. Herein, we seek to unveil potential molecular cell surface markers on t(4;14) MM.

**Figure 1:**
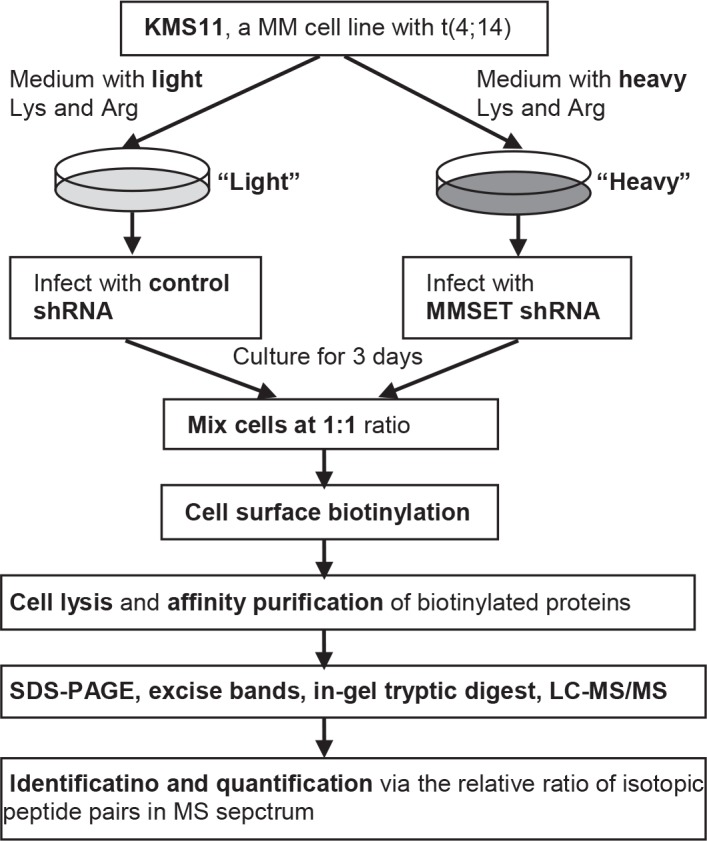
Schematic showing the experiment strategy for the relative quantification of cell surface membrane proteins KMS11 cells were cultured in medium with either light Lys and Arg (^12^C_6_ L-Lycine and ^12^C_6_^14^N_4_ L-Arginine) or heavy Lys and Arg (^13^C_6_ L-Lycine and ^13^C_6_^15^N_4_ L-Arginine) for a minimum of 6 doubling times, and then treated with control shRNA or MMSET shRNA. After 3 days, cells were mixed at 1:1 ratio and enriched cell surface protein fractions were prepared by cell surface biotinylation and NeutrAvidin-affinity chromatography. The enriched membrane proteins were analyzed by SDS-PAGE. The entire gel lane was segmented into approximately 20 sections, followed by in-gel tryptic digestion. Peptide extracts were analyzed by nanoLC Orbitrap-MS, and the protein precursors were identified using Mascot Server. Finally relative quantification of protein expression was determined by the chromatographic response observed for each isotopic peptide pair in the MS.

Plasma membrane proteins are present in relatively low abundance and therefore are often overlooked or not identified in broad spectrum, whole cell, or tissue arrays [15]. Prefractionation enrichment strategies are critical for comprehensive profiling of the plasma membrane proteome. A number of methodologies have been adopted, including the use of a membrane-impermeable biotinylation reagent that selectively labels cell surface proteins which are subsequently purified by avidin-affinity chromatography [16, 17]. Recently stable isotope labeling by amino acids in cell culture (SILAC) has become widely used in mass spectrometry (MS)-based quantitative proteomics [16-19]. This is proving to be a simple, robust, yet powerful method to complement transcription measurements at the protein level. In this study, we identified differentially expressed plasma membrane using cell surface biotinylation and avidin affinity chromatography combined with a SILAC-based MS approach (Figure 1). Our results illustrated that SLAMF7 might be a novel cell surface biomarker associated with t(4;14) MM.

## RESULTS

### Cell Surface Protein Enrichment and Identification by MS Analysis

In order to identify cell surface biomarkers associated with t(4;14) MM, we cultured KMS11, a t(4;14) MM cell line, with SILAC method. The “light” and “heavy” cell populations were treated with control or MMSET shRNAs respectively, then a cell line pair was generated (Supplementary Fig. S1). The cell line pair were mixed at 1:1 ratio by cell counting, and labeled with sulfo-NHS-LC-biotin in situ. The labeled cells were lysed and plasma membrane proteins were enriched by avidin affinity chromatography followed by separating the elution on SDS-PAGE. Extracted peptides from in-gel trypsin digested gel slices were subjected to nanoLC Orbitrap-MS analysis. MS data were analyzed using MaxQuant software (Fig. 1). After MS analysis, we identified 144 differentially expressed proteins with P (ratio significance B) <0.05 (Table 1 and Supplementary Table S1). The cellular localization of each identified protein was further investigated based on Gene Ontology Annotation (GOA) database (http://www.ebi.ac.uk/GOA). Approximately 51% of identified proteins are membrane or membrane-associated proteins (Supplementary Fig. S2). Known functions of some of these proteins include cell-cell or cell-matrix adhesion, receptors of cytokines or growth factors, and transporter of substances across membranes.

**Table 1:** Representative cell surface proteins potentially associated with MMSET overexpresion in t(4;14) MM based on quantitative mass spectrometry

Protein Names	Sequence Coverage [%]	Ratio H/L	Ratio H/L Normalized	Ratio H/L Significance	Ratio H/L Count
FGFR3	23	0.3897	0.58153	0.0014683	48
IL6ST	3.6	0.37561	0.55736	0.00066049	3
CD99L2	12.9	0.3916	0.53213	0.00026018	6
SLAMF7	11.9	0.25042	0.34509	1.95E-09	9

H, KMS11 with “heavy” isotopes treated with shMMSET; L, KMS11 with “light” isotopes treated with control shLuc.

### Validation of Differential Protein Expression

**Figure 2:**
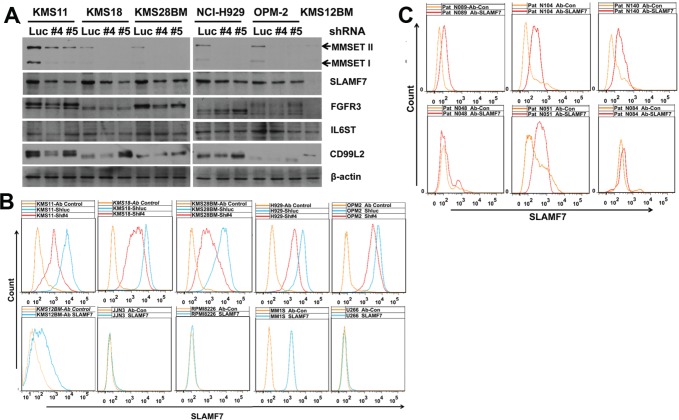
Validation of LC-MS/MS measurements with selected proteins T(4;14) MM cell lines were treated with MMSET shRNAs for 3 days. (A) Whole cell lysates were prepared from infected cells and protein levels were analyzed by Western-blot. b-actin was used as an internal control. KMS12BM is a non-t(4;14) MM cell line, others are t(4;14) MM cell lines. (B) Determination of SLAMF7 expression in MM cell lines by indirect flow cytometric analysis. Ab control, isotype mouse IgG. ShLuc, control shRNA; Sh#4 and Sh#5, MMSET shRNAs. KMS11, KMS18, KMS28BM, NCI-H929 and OPM-2 are t(4;14) positive, other cell lines are t(4;14) negative. Results indicated SLAMF7 was universally over-expressed in t(4;14) MM cell lines and down-regulated by MMSET knockdown treatments. (C) Determination of SLAMF7 expression in primary MM cells by direct flow cytometric analysis. Ab control, mouse IgG2b-FITC. Patient samples N089, N104 and N140 are t(4;14) positive, and patient samples N048, N051 and N084 are t(4;14) negative.

To validate the findings of above MS-based quantification results, we analyzed selected cell surface proteins by Western-blot. SLAMF7, identified by MS analysis as one of most differentially expressed proteins, was selected for validation because it is currently being tested as a potential therapeutic antibody target in MM [20]. Three more differentially expressed proteins were also selected for further analysis: FGFR3, which is known to be associated with t(4;14) MM [8]; IL6ST, a signal transducer shared by many cytokines including interleukin 6 [21]; CD99L2, a cell-surface protein that is similar to CD99 [22]. Western-blot analysis indicated only SLAMF7 was over-expressed in t(4;14) MM cell lines and decreased by MMSET knockdown (Fig. 2A). FGFR3, IL6LT and CD99L2 were also over-expressed in t(4;14) MM cell lines, but they might not be associated with MMSET overexpression as their expression was not changed with MMSET knockdown, except that FGFR3 was reduced in KMS28BM cells and IL6LT was reduced in KMS18 cells (Fig. 2A). We further validated SLAMF7 expression by flow cytometric analysis, and confirmed that SLAMF7 was over-expressed in t(4;14) MM cell lines and reduced upon MMSET knockdown (Fig. 2B), while four of five non-t(4;14) MM cell lines expressed low level of SLAMF7 (Fig. 2B). Direct flow cytometric analysis indicated that SLAMF7 was expressed highly in all of three t(4;14) patient samples and one of three non-t(4;14) patient samples, and weakly in two of three non-t(4;14) patient samples (Fig. 2C). In summary all the t(4;14) MM cell lines and patient samples tested over-expressed SLAMF7 whereas only some of the t(4;14) negative cell lines and patient samples over-expressed SLAMF7. The data presented here suggest that SLAMF7 over-expression might be associated with MMSET expression in t(4;14) MM.

### MMSET Regulating the Transcription Level of SLAMF7 Gene

**Figure 3:**
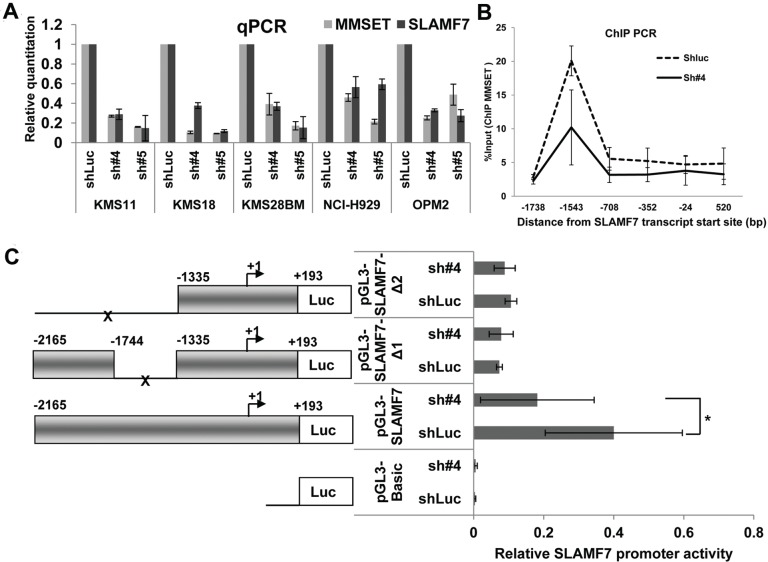
MMSET regulated the transcription level of SLAMF7 gene (A) Cells were treated with shRNAs and cultured for 48 h. QPCR analysis showed SLAMF7 transcription was reduced greatly upon MMSET knockdown. Columns, mean; bars, SD. Average C_T_ values were first normalized against the housekeeping gene b-*Actin* and converted to the induced fold change relative to the vehicle control. (B) MMSET binding was concentrated upstream of the SLAMF7 promoter (near −1,500 bp). Quantitative ChIP assay indicated that MMSET occupied the 5' end promoter region of SLAMF7 gene in KMS11 cells, while the MMSET occupancy was absent in KMS11/MMSET knockdown cells. MMSET Ab, rabbit IgG against MMSET protein C-terminus. (C) Luciferase reporter assay on SLAMF7 promoter in KMS11 cells. The left panel shows a schematic drawing of various fragments of the SLAMF7 promoter in the luciferase reporter vectors. The promoter length is indicated by the numbering relative to +1 position of transcription start site. Reporter constructs were transfected into KMS11 cells and assayed for luciferase activity. Luciferase activity was normalized to a co-transfected Renilla luciferase reporter. Data represent the mean ± SD derived from 3 separate experiments. * indicates *p*<0.05, ** indicate *p*<0.01. ShLuc, control shRNA; Sh#4 and Sh#5, MMSET shRNAs.

To explore the mechanism of MMSET protein regulating the expression of SLAMF7, we determined SLAMF7 mRNA levels upon MMSET knockdown. Quantitative PCR (qPCR) analysis indicated that SLAMF7 mRNA levels were significantly reduced upon MMSET knockdowns in all t(4;14) MM cell lines (Fig. 3A). We performed quantitative Chromatin Immunoprecipitation (ChIP) for MMSET II occupancy at the 5' regulatory region of the SLAMF7 gene. We observed that the MMSET II protein binding was concentrated in an upstream region (near −1,500 bp) of *SLAMF7* transcript start site. We also found a markedly reduced presence of the MMSET II protein in the *SLAMF7* promoter when MMSET expression was knocked-down (Fig. 3B). We further performed luciferase reporter assay using the *SLAMF7* promoter (−2165/+193) and its two truncations in KMS11 cells. We found the activity of *SLAMF7* promoter (−2165/+193) was decreased significantly when MMSET expression was reduced. Furthermore, deletion of the −1744/−1335 region greatly reduced *SLAMF7* promoter activity (approximately four-fold) (Fig. 3C). These results suggest that MMSET is an important functional element for *SLAMF7* promoter activity and is likely to reside in the −1744/−1335 region of *SLAMF7* promoter.

### Characterization of the SLAMF7 Activity in T(4;14) MM Cells

**Figure 4:**
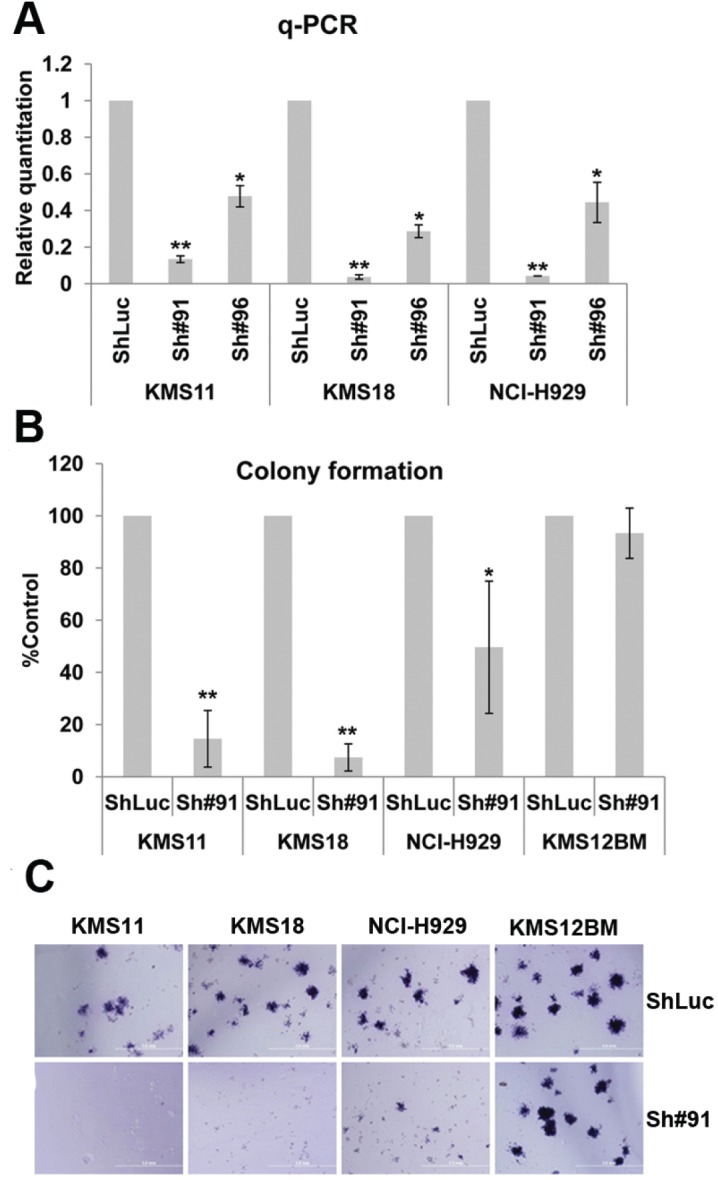
Characterization of the SLAMF7 activity in t(4;14) MM cells by colony formation assay in methylcellulose media (A) Cells were treated with shRNAs and cultured for 48 h. QPCR analysis showed SLAMF7 transcription was reduced greatly upon shSLAMF7 treatment. (B and C) T(4;14) MM cells with SLAMF7 ShRNA treatment had markedly reduced ability to form colonies compared with control shLuc. Data represent the mean ± SD derived from 3 separate experiments. KMS12BM, a non-t(4:14) MM cell line with low level of SLAMF7, is a negative control. ShLuc, control shRNA; Sh#91, and Sh#96, SLAMF7 shRNAs.

To investigate the function of SLAMF7 in t(4;14) MM cells, its expression was knocked down with SLAMF7 shRNA. SLAMF7 down-regulation was confirmed by qPCR and flow cytometric analysis (Fig. 4A and Supplementary Fig. S3A). Cell cycle analysis indicated that the knocked down of SLAMF7 decreased S phase cells and induced G1 arrest or apoptosis in t(4;14) MM (Supplementary Fig. S3B). To assay for clonogenetic capacity, MM cells were cultured in methylcellulose medium. Eleven days after cell plating, t(4;14) MM cells with SLAMF7 shRNA treatment had markedly reduced ability to form colonies compared with controls, evidenced by decreased colony number and size (Fig. 4B and 4C). In the above assay, SLAMF7 shRNA had little effect on KMS12BM, a non-t(4:14) MM cell line with low level of SLAMF7.

## DISCUSSION

MM is a malignant plasma cell disorder that accounts for approximately 10% of all hematologic tumors [23]. The t(4;14) translocation is the second most common translocation in MM, affects 15% of MM patients, and is associated with the worst prognosis [4, 5, 24, 25]. The t(4;14) translocation results in over-expressing *MMSET* genes [8]. Recently MMSET has been shown to have histone methyltransferase activity and knockdown studies have demonstrated that MMSET upregulation contributes to cellular adhesion, clonogenic growth and tumorigenicity [1, 10, 11]. Although MMSET proteins are likely to be associated with the pathogenesis of t(4;14) MM, currently there is no drug targeting MMSET proteins directly. The proteins closely associated with or regulated by MMSET expression might potentially constitute novel biomarkers or therapeutic targets for t(4;14) MM. Because of membrane proteins’ accessibility, they constitute about 60% of approved drug targets [26]. However, the relative low abundances and hydrophobicity of most membrane proteins impose challenges for determining their identities. In this study, we identified cell surface markers depending on MMSET expression using cell surface biotinylation and avidin affinity chromatography combined with a SILAC-based MS approach. MS results indicated that SLAMF7 might be a novel cell surface biomarker associated with t(4;14) MM.

SLAMF7 (also known as CS1, CD319 and CRACC), one of SLAM (signaling lymphocytic activation molecule) family members, is a transmembrane glycoprotein with 355 amino acids [27]. The cytoplasmic domain of SLAMF7 contains two immunoreceptor tyrosine-based switch motifs (ITSMs), whose activation could induce tyrosine phosphorylation of ERK1/2 [27, 28]. SLAMF7 is selectively expressed on activated B cells, NK cells, mature dendritic cells and a subset of cytotoxic T cells, and a positive regulator of above cells [27, 29-31]. Recently, it was reported that SLAMF7 is over-expressed on MM cells and a potential therapeutic target for the treatment of MM, but the mechanism of over-expressing SLAMF7 on MM is not known [20, 32]. In previous gene expression analysis in MM patient samples, t(4;14) MM cells have one of the highest SLAMF7 expression [32]. Our data also showed that SLAMF7 was over-expressed in all of three t(4;14) primary MM samples and one of three non-t(4;14) primary MM samples. In this study we found MMSET knockdown could reduce SLAMF7 levels in t(4;14) MM cells. Furthermore, we found MMSET proteins were enriched in the SLAMF7 promoter region and regulated the transcription level of SLAMF7. Thus, one of the mechanism leading to SLAMF7 expression in MM and the higher expression observed in t(4;14) MM may be a direct effect on it transcription and expression by the MMSET protein. Previous studies showed that SLAMF7 could induce proliferation and autocrine cytokine expression on human B lymphocytes [31]. Here we showed that SLAMF7 knockdown could induce cell cycle G1 arrest or apoptosis, and reduce colony formation in t(4;14) MM cells, suggesting a potential role of SLAMF7 in the tumorigenicity of MM. Since the cell cycle plays a critical role in chemosensitivity for combination chemotherapy [33], we also investigated whether SLAMF7 knocking down could potentiate the activity of bortezomib, a proteasome inhibitor with significant activity in myeloma. Although previous report indicated bortezomib could enhance Elotuzumab (a humanized monoclonal antibody against SLAMF7) mediated antibody-dependent cell-mediated cytotoxicity [34], our data indicated SLAMF7 knocking down only marginally potentiated bortezomib (Supplementary Fig. S4).

Sequence-specific gene silencing with small interfering RNA (siRNA) has transformed basic science research, and the efficacy of siRNA therapeutics toward a variety of diseases is now being evaluated in pre-clinical and clinical trials [35]. The key therapeutic advantage of using siRNA lies in its ability to specifically and potently knock down the expression of disease-causing genes of known sequence. However, clinical use of siRNAs encounters one of obstacles: delivery of siRNAs to the appropriate cells. Antibody-mediated delivery is an effective method of targeting siRNA to particular cells [36]. Our study showed that SLAMF7 overexpression in t(4;14) MM was associated with MMSET expression. Thus, it is potential to develop t(4;14) MM targeted therapy by SLAMF7 antibody mediated MMSET siRNA delivery. This therapeutic strategy will achieve two levels of targeting for t(4;14) MM: tumor cell selective delivery by the SLAMF7 antibody and gene pathway selectivity by the MMSET siRNA. Therefore, this therapeutic strategy would avoid nonspecific silencing and toxicity in bystander cells.

## MATERIALS AND METHODS

### Cell Lines and Cell Culture

Human MM cell lines KMS11, KMS12BM, KMS18, KMS28BM and OPM-2 were maintained in RPMI 1640, supplemented with 10% fetal calf serum (FCS), 100 U/mL penicillin and 100 μg/mL streptomycin. MM cell line NCI-H929 was cultured in RPMI 1640 with 15% FCS and 0.00036% 2-mercaptoethanol. All MM cell lines except KMS12BM are t(4;14) positive. All MM cell lines are from Prof Rafael Fonseca and P. Leif Bergsagel laboratories (Mayo Clinic, Scottsdale) and have been tested, authenticated, and previously used in the peer-reviewed articles from the laboratories [37, 38]. HEK293T cells (from ATCC, Manassas, VA) were maintained in DMEM, supplemented with 10% FCS, 100 U/mL penicillin and 100 μg/mL streptomycin. All cells were grown at 37 °C in a humidified atmosphere with 5% CO_2_.

### SILAC

SILAC was performed as described previousely [39]. Two populations of KMS11 cells were maintained in lysine and arginine depleted RPMI1640 medium (Pierce SILAC Protein Quantitation Kit) supplemented with 10% dialyzed FCS, 100 U/mL penicillin and 100 μg/mL streptomycin. The “light” cell population was supplemented with normal L-lysine and L-arginine, and the “heavy” cell population was supplemented with^13^C_6_-L-lysine and ^13^C_6_^15^N_4_-L-arginine. The cells were allowed to grow for six doubling times to achieve maximum incorporation of heavy amino acids into proteins. Labeling efficiencies were determined by MS analysis of peptides from light- and heavy-labeled proteins and were found to be >98%.

### Virus Production and Infection

The sequences encoding short hairpin RNAs (shRNAs) against MMSET or SLAMF7 (Supplementary Table S2) were cloned into lentiviral vector pLKO.1. Lentivirus infection was performed as previously described [40, 41]. Briefly, lentiviruses were produced by transiently cotransfecting HEK293T cells with the shRNA-expressing lentivirus vector, packaging plasmid pMDLg/pRRE and pRSV-Rev, and VSV-G envelope plasmid pCMV-VSVG using X-tremeGENE HP DNA Transfection Reagent (Roche Diagnostics) according to the manufacturers’ instructions. Cells were infected with lentiviruses in the presence of 8 µg/mL polybrene (Sigma-Aldrich). Protein expression was analyzed 3 days after infection.

### Cell Surface Protein Isolation

“Heavy” KMS11 cells were treated with MMSET shRNA and cultured for 72 h, while “light” KMS11 cells were treated with shLuc as a control. The two cell populations were counted and mixed in a 1:1 ratio (a total of 4x10^7^ cells) followed by two rounds of washing in PBS. Cell surface protein isolation was performed using Pierce Cell Surface Protein Isolation Kit (#89881) according to the manufacturer's protocol. Briefly, cells were re-suspended at 1x10^6^ cells/mL in PBS with 0.25 mg/mL Sulfo-NHS-SS-Biotin and incubated for 30 min at 4°C. The biotinylation reaction was quenched by the addition of 2 mL of Quenching Solution following two washes with TBS. Cells were lysed in Lysis Buffer containing protease inhibitors (Halt Protease Inhibitor Cocktail, Pierce #78410). Cell debris was removed by centrifugation at 10000 × g for 2 min at 4°C. Biotinylated proteins were affinity purified with NeutrAvidin Agarose and eluted with SDS-PAGE sample buffer (62.5 mM Tris HCl, pH 6.8, 1% SDS, 10% glycerol, 50 mM DTT).

### Mass Spectrometry Analysis

Eluted proteins were separated by one-dimensional 4-12% NuPageNovexBis-Tris Gel (Invitrogen), stained using the Colloidal Blue Staining Kit (Invitrogen) and cut into 17 bands followed by in-gel digestion. . In-gel tryptic digestion, peptide extraction and reconstitution were carried out as previously described[42]. Vacuum dried peptide samples were reconstituted in 0.1% formic acid and analysed using nanoHPLC (Proxeon, Thermo Scientific) coupled to a LTQ Orbitrap XL (Thermo Scientific). Peptides were trapped onto a C18 pre-column and separated on an analytical column using 2% acetonitrile/0.1% formic acid as Solvent A and 80% acetonitrile/0.1% formic acid as Solvent B. A 120 min gradient ranging from 5% to 50% solvent B, followed by a 5 min gradient ranging from 50% to 100% Solvent B at the flow rate of 250 nL/min was used. Peptide samples were analyzed on an Orbitrap XL (Thermo Scietific) with two technical replicates. Survey full scan MS spectra (m/z 300–1400) were acquired with a resolution of r = 60,000 at m/z 400, an AGC target of 1e6, and a maximum injection time of 500 ms. The ten most intense peptide ions in each survey scan with an ion intensity of >2000 counts and a charge state ≥2 were isolated sequentially to a target value of 1e4 and fragmented in the linear ion trap by collisionally-induced dissociation using a normalized collision energy of 35%. A dynamic exclusion was applied using a maximum exclusion list of 500 with one repeat count, repeat, and exclusion duration of 30 s.

### Identification and Quantification of Peptides and Proteins

Data were searched using Mascot (version 2.2; Matrix Science, London, UK) against a concatenated target/decoy database, prepared appending a sequence-reversed by human International Protein Index (IPI) (version 3.52, 73,928 sequences) and adding common contaminants such as human keratins, porcine trypsin, and proteases to yield a total of 148,380 sequences. Database searches were performed with tryptic specificity allowing maximum two missed cleavages and two labeled amino acids as well as an initial mass tolerance of 7ppm for precursor ions and 0.5Da for fragment ions. Cysteine carbamidomethylation was searched as a fixed modification, and N-acetylation and oxidized methionine were searched as variable modifications. Labeled arginine and lysine were specified as fixed or variable modifications, depending on the prior knowledge about the parent ion. SILAC peptide and protein quantification was performed with MaxQuant version 1.0.13.13 (19, 20) using default settings. Maximum false discovery rates were set to 0.01 for both protein and peptide. Proteins were considered identified when supported by at least one unique peptide with a minimum length of six amino acids. The differentially expressed proteins were selected based on the Ratio Significance B < 0.05 [43].

### Immunoblot Analysis

Cells were treated with shRNAs and cultured for 72 h. Cells were collected and lysed in RIPA buffer as described previously [44]. Equal amounts of protein were separated on SDS–polyacrylamide gels and transferred to PVDF membranes. The blots were probed with antibodies against CS1(SLAMF7) (sc-53576), FGFR3 (sc-13121) and β-Actin (sc-69879) were from Santa Cruz Biotechnology. The blots were probed with antibodies against IL6ST (#H00003572-M05, NOVUS), MMSET (#75359 Abcam), CD99L2 (#AF5185, R&D SYSTEMS). Western Blotting Luminol Reagent (sc-2048) was used for detection on film from Santa Cruz Biotechnology.

### Preparation of primary myeloma cells

Primary myeloma cells were obtained from bone marrow aspirates by purification with human CD138 selection kit (StemCell Technologies, #18357). Purified primary myeloma cells were re-suspended with RPMI/DMSO freezing medium and stored in liquid nitrogen. All primary cells were obtained from routine diagnostic samples after informed consent was provided by the patients and permission given by the NUS Institutional Review Board.

### Flow Cytometric and Cell Cycle Analysis

Cells were treated with shRNAs and cultured for 72 h. Then, cells were collected and stained for flow cytometric analysis as previously described [45]. Primary antibody was CS1(SLAMF7) (sc-53576, Santa Cruz Biotechnology), and secondary antibody was Alexa Fluor 488 goat anti—mouse IgG (Invitrogen). Directly conjugated CS1 antibody (FITC, sc-53577, Santa Cruz Biotechnology) was used for patient sample analysis. The data were analyzed using FlowJo (Tree Star, Inc.). Cells were treated with SLAMF7 shRNA and cultured for 72 h. Then, cells were collected and stained with propidium iodide (PI) for cell cycle analysis as previously described [46].

### QPCR

Cells were treated with shRNAs and cultured for 48 h. Total RNA was extracted by using the Qiagen RNeasy Mini kit (Germany). Using oligo(dT)_20_ primer and reverse transcriptase, cDNA was created by using the Invitrogen SuperScript III First-Strand Synthesis System. QPCR analysis was performed as previously described [41]. The primers for qPCR were listed in Supplementary Table S3.

### ChIP Assay

KMS11 cells were treated with shRNAs for 72 h and fixed in 1% formaldehyde. ChIPs were performed using SimpleChIP™ Enzymatic Chromatin IP Kit (#9002, Cell Signaling, Danvers, MA) according to the manufacturer's protocol. A rabbit anti-MMSET antibody for ChIP was produced from a rabbit immunized against the recombinant GST-tagged C-terminal peptides of MMSET II (residues 1329–1365), which was expressed in *Escherichia coli* and purified in accordance with the manufacturer's instructions (GE Healthcare). Input controls consisted of 2% chromatin before immunoprecipitation. Purified DNA from cross-linked cells was used for qPCR with a subset of primers across *SLAMF7* promoter (Supplementary Table S4). Data obtained by qPCR were plotted as percent input control.

### Construction of luciferase reporter vectors

The *SLAMF7* promoter region was PCR amplificated from KMS11 genomic DNA using forward and reverse primers containing *Xho* I and *Hin*d III restriction sites (Supplementary Table S5). After restriction digestion, the fragments were cloned into the pGL3-Control vector (digested with *Xho* I and *Hin*d III to delete *SV40* promoter) to generate the *SLAMF7* promoter constructs, pGL3-*SLAMF7* (-2165/+193) and pGL3-*SLAMF7*-Δ2 (−1335/+193). The pGL3-*SLAMF7*-Δ1 (deletion of -1744/-1336) was generated by cloning *SLAMF7* -1335/+193 into pGL3-*SLAMF7* (-2165/+193) (digested with *Exo*R I and *Hin*d III to delete -1733/+193). The constructs were verified by nucleotide sequencing.

### Luciferase Assay

KMS11 cells were treated with shRNAs and cultured for 24 h. Then, cells were cotransfected with firefly luciferase reporter construct and Renilla luciferase reporter vector pRL-SV40 (Promega) in a ratio of 200:1 using X-tremeGENE transfection reagent (Roche Diagnostics). Cells were harvested after 24 h and analyzed for luciferase activity using Dual-Luciferase Reporter Assay System (Promega). The promoter activity was calculated by dividing the luciferase activity of constructs by the internal Renilla luciferase activity.

### Methylcellulose Colony Formation Assay

Methylcellulose media (H4230, MethoCult^®^, StemCell Technologies) consisted of 1% methylcellulose, 30% FCS, 100 U/mL penicillin and 100 μg/mL streptomycin, and 1% BSA. Colony formation assay was performed as our previously described [41].

### Statistical Analysis

Results were expressed as mean values ± SD, and t-test was used for evaluating statistical significance. Results were considered to be significant when *p* < 0.05.

## SUPPLEMENTARY TABLE AND FIGURES




