# Supplementary material for: Plasma Membrane Proteomics Identifies Biomarkers Associated with MMSET Overexpression in T(4;14) Multiple Myeloma
Source: Oncotarget. 2013 Jun 26;4(7):1008–18. doi: 10.18632/oncotarget.1049 (PMC3759662; doi:10.18632/oncotarget.1049)
Supplement: Supplementary file 2 [file oncotarget-04-1008-s002.doc]

Plasma Membrane Proteomics Identifies Biomarkers Associated with MMSET Overexpression in T(4;14) Multiple Myeloma – Xie et al


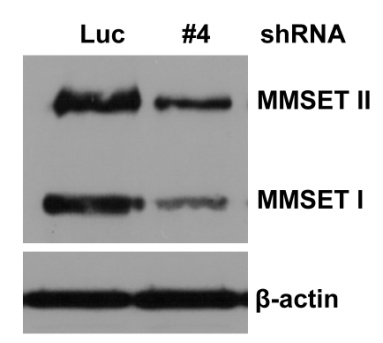


Supplementary Figure S1: The KMS11 cell line pair for plasma membrane proteomics analysis. KMS11cells were cultured with light and heavy SILAC medium respectively. “Heavy” KMS11 cells were treated with MMSET shRNA and cultured for 72 h, while “light” KMS11 cells were treated with shLuc as a control. The two cell populations were counted, then 2x10^6^ cells from each population were lysed for Western-blot analysis, and 2x10^7^ cells from each population were mixed (1:1 ratio) for plasma membrane protein enrichment and proteomics analysis.


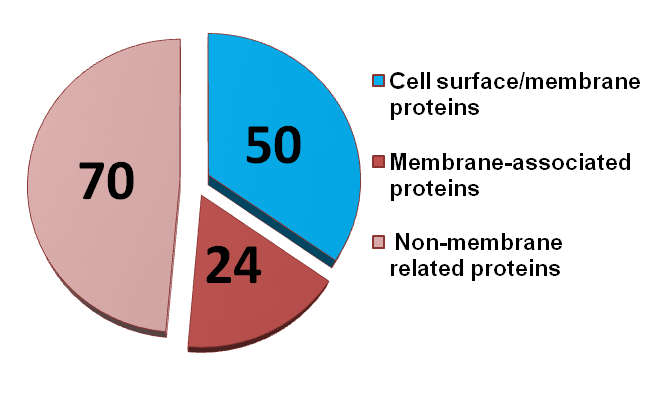


Supplementary Figure S2: Pie chart of subcellular classification of the 144 differential proteins identified from KMS11 and KMS11/KD MMSET cells. From the LC-MS/MS results, we identified 144 proteins differentially expressed in KMS11 and KMS11/MMSET knockdown. The cellular localization of each identified protein was further investigated based on Gene Ontology Annotation (GOA) database (<http://www.ebi.ac.uk/GOA>). Plasma membranes identified accounted for 35% of total proteins, which is similar to the previous analysis (Journal of Biomolecular Techniques V21:108–115).

**(A)**


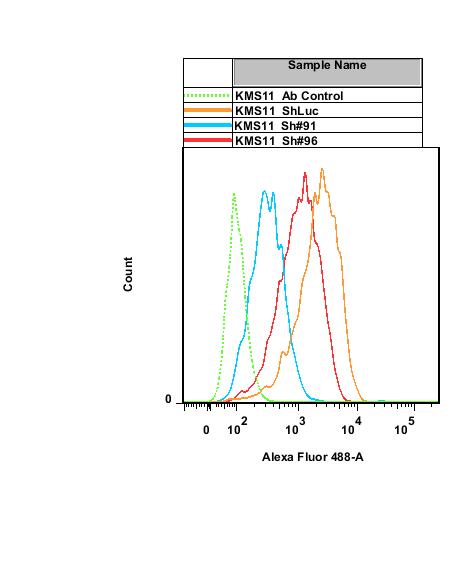

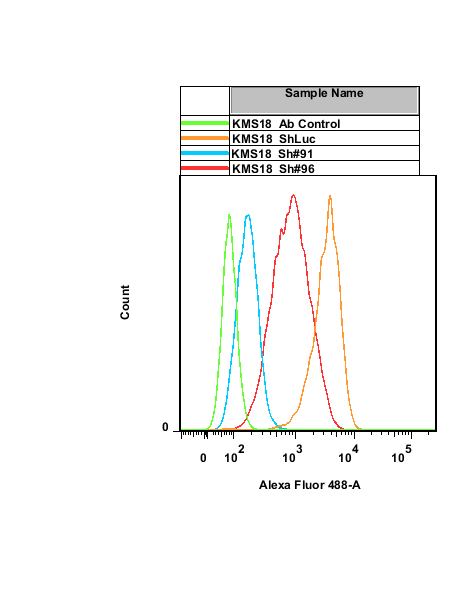

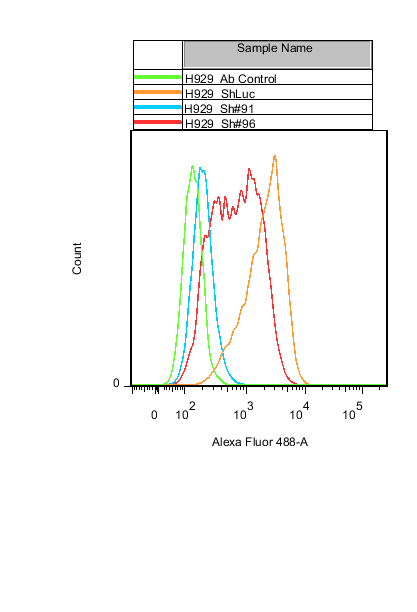


**Count**

**SLAMF7**

**(B)**


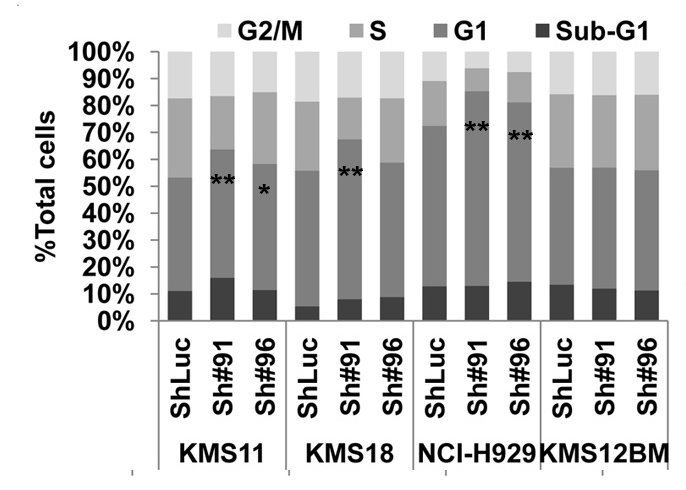


Supplementary Figure S3: Characterization of the SLAMF7 activity in t(4;14) MM cells. (A) Cells were treated with shRNAs and cultured for 48 h. Flow cytometric analysis showed SLAMF7 expression were reduced greatly upon shSLAMF7 treatment. Ab control, isotpye mouse IgG. (B) Cell cycle analysis indicated that the knocking down of SLAMF7 decreased cell S phase and induced G1 arrest or apoptosis in t(4;14) MM cell lines. Data represent the mean derived from 3 separate experiments. ShLuc, control shRNA; Sh#91 and Sh#96, SLAMF7 shRNAs. * indicates *p*<0.05, ** indicate *p*<0.01.


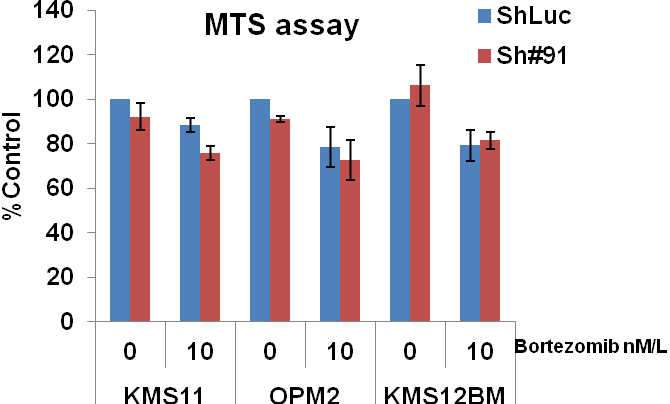


Supplementary Figure S4: Antimyeloma activity of combination of Bortezomib (Velcade) and SLAMF7 knockdown. Cells were co-treated with Bortezomib and SLAMF7 shRNA and incubated for 48 h. The proliferation was determined by MTS colorimetric assay (Promega). Data represent the mean ± SD derived from 2 separate experiments with triplicate wells per condition. ShLuc, control shRNA; Sh#91, SLAMF7 shRNA.
